# Understanding beliefs related to physical activity in people living with axial Spondyloarthritis: a theory-informed qualitative study

**DOI:** 10.1186/s41927-022-00270-2

**Published:** 2022-07-25

**Authors:** Anne-Kathrin Rausch Osthoff, Irina Nast, Karin Niedermann

**Affiliations:** 1 School of Health Sciences, Institute for Physiotherapy, Zurich University of Applied Sciences, Winterthur, Switzerland; 2 Department of Orthopaedics, Rehabilitation and Physical Therapy, Leiden University Medical Centre, Leiden, Netherlands

**Keywords:** Physical activity, Cardiorespiratory training, Exercise, Beliefs, Attitudes, Theory of planned behaviour, Axial Spondyloarthritis

## Abstract

**Background:**

People living with axial Spondyloarthrtis (axSpA) have an increased risk of cardiovascular diseases, which can be reduced by regular physical activity (PA) and its subset of cardiorespiratory training (CRT). To fulfil their crucial role in PA promotion, physiotherapists and other health professionals need to understand the beliefs that people living with axSpA possess concerning general PA and CRT. The aim of this study is to explore these behavioural, normative and control beliefs.

**Methods:**

A qualitative descriptive design approach was chosen. Five semi-structured focus group interviews with 24 individuals living with axSpA were performed. Data was analysed using structured thematic qualitative content analysis.

**Results:**

People with axSpA possessed multifaceted behavioural, normative and control beliefs concerning general PA and CRT. Behavioural beliefs revealed a positive attitude towards general PA, with participants mentioning numerous physical, psychological, and social benefits and only few risks. However, the conceptual difference between general PA and CRT, and the relevance of CRT, was unclear to some participants. Normative beliefs were expressed as the beliefs of significant others that influenced their motivation to comply with such beliefs, e.g. spouses, other people living with axSpA, rheumatologists. Regarding control beliefs, general PA and CRT were both mentioned as effective self-management strategies to control the disease. From experience, a high level of self-discipline, as well as technology, were shown to be useful.

**Conclusions:**

General PA is understood to be an important self-management strategy for people with axSpA and most participants build general PA into their daily routines. They believe that general PA beneficially impacts personal health and wellbeing. However, some participants are unaware of the difference between general PA and CRT and the important impact that this difference could have on their health. The consequences of CRT promotion for people living with axSpA should be the subject of further research.

**Supplementary Information:**

The online version contains supplementary material available at 10.1186/s41927-022-00270-2.

## Background

Axial Spondyloarthritis (axSpA) is a chronic, inflammatory rheumatic disease that leads to structural impairments and functional limitations [1]. AxSpA increases the risk of cardiovascular diseases [2, 3] and has an impact on flexibility [4], balance [5], muscle strength [6] and cardio-respiratory capacity [7].

As the cornerstones of disease management, exercise and effective drug treatment are recommended [1, 8]. Correspondingly, the EULAR (European Alliance of Association for Rheumatology) physical activity (PA) recommendations for individuals with rheumatic and musculoskeletal diseases (RMDs) [9] strongly emphasize the importance of PA promotion. Evidence shows that exercising, according to the public health recommendations for health-enhancing PA, is effective, safe, and feasible for individuals with RMDs [10], and that it should be performed throughout the course of the disease [9]. Exercise, particularly cardio-respiratory training (CRT), can be effective medicine reducing cardiovascular risk when the dose, i.e. intensity, duration and frequency, is adequately applied [11–14]. Current disease management recommendations state that patients should be continuously integrated into exercise programs, rather than taking rest or inactive periods during or after a flare up [9, 15].

Despite existing evidence, individuals with axSpA are often physically active at low-to-moderate intensity levels and spend less time performing vigorous PA than healthy people [16, 17]. In Switzerland, although 88% of individuals with axSpA are willing to improve their fitness, almost 80% are unaware of the increased risk of cardiovascular diseases [18]. The best facilitator of general PA is to build intrinsic motivation, guided by enjoyment and personal interest [19]. The most commonly reported barriers to general PA are disease-specific symptoms, notably fatigue and pain [20]. Previous research has shown that the most frequent facilitators, particularly to CRT, are knowledge, homogenous group composition, and high perceived motivation. Contrarily, the most frequent barriers to CRT are lack of motivation or information, hindering disease symptoms, and problem of timing in the daily routine [21].

The EULAR recommendations on PA state that all health professionals should provide advice on PA, however, interventions should be delivered by health professionals competent in PA principles and RMDs [9]. Physiotherapists (PTs) are experts in PA promotion [22] and support people with RMDs to live an active lifestyle and exercise at a correct dose. PTs need to be precise in their instructions and use clear definitions and terms to achieve this. There is a difference between the concepts of being “physically active” and “exercising” [23], which often becomes blurred in communication with patients. Inconsistencies arise through the over-reporting or under-reporting of behavioural activity because the understanding of terms varies between the individuals involved [23].

PA is defined as, ‘‘any bodily movement produced by skeletal muscles that results in energy expenditure above resting levels. PA broadly encompasses exercise, sports, and physical activities done as part of daily living, occupation, leisure, and active transportation” [24]. Exercise is a subcategory of ‘‘PA that is planned, structured, and repetitive and [that] has, as a final or intermediate objective, the improvement or maintenance of one or more dimensions of physical fitness” [25]. Public health recommendations for health-enhancing PA encompass four exercise dimensions (aerobic, strength, neuromotor, flexibility) [24].

**Fig. 1:**
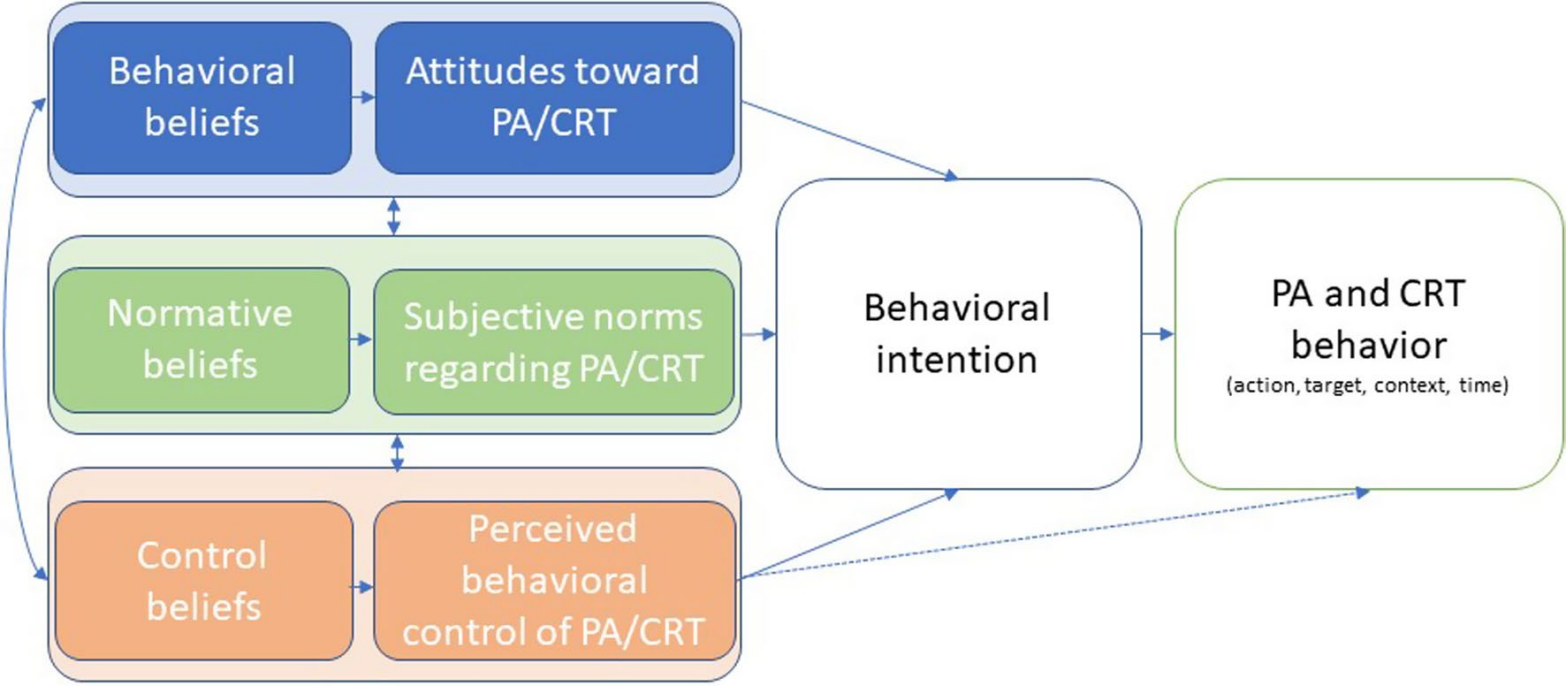
Adapted pathway of theory of planned behaviour [29]. *PA* physical activity, *CRT* cardiorespiratory training

Numerous theoretical models have been applied that aim to change PA behaviour in people with RMDs [26, 27]. Changes in awareness, beliefs, attitudes, motivation or knowledge may influence our PA behaviour. These psychological constructs are not open to direct observation, but models, such as the Theory of Planned Behaviour (TPB), support research in this field. The TPB has been used widely in research on PA behaviour (see Fig. 1). It postulates that behaviour (e.g. PA) depends on intention (motivation) and ability (behavioural control) [28]. In this respect, three constructs are relevant: (1) attitude toward the behaviour (a function of behavioural beliefs), defined as ‘’the psychological tendency that is expressed by evaluating a particular entity with some degree of favour or disfavour’’; (2) subjective norm (arising from normative beliefs), defined as ‘’the beliefs of significant others and the extent that one wishes to comply with such beliefs of [other] people’’; (3) perceived behavioural control (a function of control beliefs), which is ‘’the perceived ease or difficulty of performing behaviour’’ and is assumed ‘’to reflect past experience as well as anticipated impediments and obstacles’’ [all citations are obtained from (Chapter 3, [28])]. An individual’s behavioural intention is determined by his or her attitude toward the behaviour, subjective norms, and perceived behavioural control. The latter is a central component in predicting behaviour: behavioural control co-determines behaviour either indirectly by shaping behavioural intention, or directly in cases where behavioural control is objectively limited by environmental circumstances.

Experience from clinical practice suggests that patients often are not aware of the distinction between PA and CRT and their specific health benefits. Therefore, the objective of this study was to explore the behavioural, normative and control beliefs concerning general PA and CRT in people living with axSpA. Understanding their beliefs would allow PTs and other health professionals to fulfil their crucial role in PA promotion.

## Methods

### Design

A qualitative research design with semi-structured focus groups of individuals with axSpA was chosen to explore patient beliefs concerning CRT and physical activity. The study applied a descriptive qualitative approach, as described by Sandelowski [30, 31]. Focus groups are a valid qualitative method to explore people’s knowledge and experiences, making use of their interaction [32, 33].

Three of the five focus groups were conceptualized primarily to learn more about the importance of PA and barriers/facilitators to CRT in individuals with axSpA [21]. The other two focus groups were used to understand more about PA behaviour and technology-based CRT. All focus group interviews were performed during the early stage of a project [34] aimed at implementing PA recommendations within an axSpA exercise group concept. The data were analysed a second time with focus on beliefs.

### Ethical considerations

This study was approved by the Ethics Commission of Canton Zurich (June 2016, BASEC-Nr. 2016-00316) and all participants gave their written informed consent. The principles of good clinical practice and the declaration of Helsinki were followed [35]. Reporting accords with the Consolidated Criteria for Reporting Qualitative Research (COREQ, Additional file 1) [36]. The characteristics of the research team are described in Additional file 2.

### Procedures/patient selection/data collection

The Ankylosing Spondylitis Association of Switzerland (Schweizerische Vereinigung Morbus Bechterew, SVMB) recruited the participants of the focus groups by sending an invitation to all their members in the German-speaking part of Switzerland. For the barrier/facilitator study [21], patients were purposefully sampled based on their self-reported PA participation levels (according to the International PA Questionnaire, short version [37]) and on their participation in a SVMB exercise group. Final selection was dependent upon achieving maximum variability of group participants in terms of sex, age, and part-time or full-time employment. For two focus groups, a convenience sample of people interested in technology-based exercising participated.

Demographic data of the participants are provided in Table 1.

**Table 1:** Characteristics of focus group participants

Characteristics	Participants (n)
Total n	24 (100%)
Gender	
Male	15 (62.5%)
Female	9 (37.5%)
Age	
Mean ± SD (range)	53 ± 14 (28–86)
Occupation	
Employed	18 (75.0%)
Retired	6 (25.0%)
Member of SVMB exercise group	8 (33.0%)
Participation in sports club	9 (37.5%)
Performance of regular CRT (at least 2x/week)	15 (62.5%)

*SD* standard deviation, *CRT* cardiorespiratory training, *SVMB* Schweizerische Vereinigung Morbus Bechterew

A few days before the focus groups took place, detailed participant information and an informed consent form were sent via e-mail to the participants. The same information was explained once again verbally at the beginning of the focus group meetings and participants gave their written informed consent. The focus group interviews took place either in a meeting room at the SVMB or at the Zurich University of Applied Sciences (ZHAW). A representative of the SVMB welcomed their participant members but were not present during the conduct of the focus group interview. The researchers explained to the participants that the aim of the study was to evaluate and further develop the SVMB exercise groups, that their opinions were of great value to the project, and that the study was independent of their engagement in the SVMB exercise groups or their current physical activity level. Furthermore, participants were assured that all data would be processed confidentially and that the transcripts of the group discussions would be anonymised. The interviewers (IN, KN, AR) were unknown to the participants prior to the study and introduced themselves, together with their professional functions at the ZHAW.

The semi-structured focus group interview guides are detailed in Additional file 3. At the beginning, participants were invited to introduce themselves with their name and, for example, age, job, recreational activities, or onset and course of disease. The interview guide contained leading and detailed open-ended questions on the following topics: appraisal of own health; personal significance of PA; and attitudes to and thoughts on PA and CRT. Three focus groups focused on the potential barriers and facilitators to PA/CRT, while two focus groups concentrated on the use of technology for PA/CRT. In addition, SVMB group exercising and expectations on the SVMB were discussed. Each focus group lasted approximately 90 min.

### Data analysis

Data was analysed using structured thematic qualitative content analysis, as described by Kuckartz [38]. The focus group audio data was transcribed and proofread by the researchers IN (psychologist) and AR (physiotherapist). Subsequently, data was imported into the Qualitative Data Analysis Software Atlas.ti [39]. Qualitative content analysis was initiated through attentive reading of the transcripts and the highlighting of important passages. Thematic main categories were defined deductively, according to the TPB’s three determinants of behaviour attitudes, norms, and perceived behavioural control, as well as their associated behavioural, normative and control beliefs. In the next step, all data were coded and quotations assigned to these three main categories. In the following step, all quotations assigned to a category were compiled. Within these, subcategories were developed inductively to describe the material within each category (IN, AR). The two researchers discussed the developed subcategories and a consensus-based detailed categorisation system was defined (Table 2). Applying this system, all the data was analysed accordingly. Finally, thematic summaries were described for all three categories of the TPB. Example quotations of patients were translated from (Swiss-)German into English.

**Table 2:** Detailed categorisation system

Category	Subcategories
Behavioural beliefs	– Conceptualization of physical activity and cardiovascular training
	– Exercise benefits
	– Exercise associated risks
Normative beliefs	– Motivation of significant others to exercise
	– Mutual commitment
	– Performance pressure
	– Image of people with axSpA
Control beliefs	– Discipline, tricks
	– Key experience
	– Limitation due to disease
	– Pain control
	– Exercise requirements in daily living (work, leisure activities)
	– Exercise opportunities in daily living (work, leisure activities)
	– Exercise opportunities offered by the SVMB
	– Expectations on course instructors
	– Support from technology

*axSpA* axial Spondyloarthritis, *SVMB* Schweizerische Vereinigung Morbus Bechterew

### Measures of trustworthiness

The trustworthiness of the study, as described by Steinke [38], has been strengthened by various measures taken during the study process: intersubjective comprehensibility was enhanced by stringent documentation of the research process, discussion of the results within the research group, and application and documentation of the categorisation system. Sampling, maximum variation strategy, as well as methods of data collection and analysis, have been chosen as indicated by the research question. Results are highlighted based on statements to allow empirical anchoring. Finally, memos were used to reflect on experiences, perceptions, and expectations of the topic, and the beliefs of the participants in terms of reflected subjectivity. Transcripts and findings were returned to participants for comments.

## Results

Results reflect the behavioural, normative, and perceived control beliefs of individuals with axSpA concerning general PA and CRT. Additional file 4: Table S3 displays example anchor quotations that underpin the following results.

### Attitudes toward behaviour: performing general PA and CRT

All participating individuals living with axSpA had a positive attitude towards PA, reporting various important benefits: physical (improving fitness, physical well-being, active control of disease); psychological (joy, self-management strategy, improvement in self-esteem, enabling a sense of achievement); and social (reinforcing exercise group, activities with healthy people or other people living with axSpA). See Additional file 4: Table S3*, e.g. “I think axSpA also has advantages. You stay active.” C6 (physical benefit); “I also feel better when I move. You have the same pain but then you know it's just a good feeling, when you don't do anything and you have pain, then it's more of a negative feeling. (..) I agree, physical activity is simply most important.” D3 (psychological); “For us, group therapy is first and foremost a social gathering. More than anything else. That's the good thing about group therapy.” E6 (social)*.

Some participants also mentioned the perceived risks of PA, such as the fear of increasing symptoms (e.g. pain) or physical overload prompting an injury or flare up. Some mentioned that they had had to discontinue their preferred sport since their diagnosis (e.g. *“I'm not allowed to do contact sports anymore.” B1).*

For many participants, regular PA was part of their self-concept (e.g. *“I am very sporty.” C6*) and an important part of their daily routine (e.g. *“I miss something if I cannot exercise.” C5)*. However, the relevance of CRT was new to some participants (e.g. *“Why are people so into cardiorespiratory training, recently, it always comes up..?” B3*) and only a few participants expressed the same positive attitude towards CRT as they did towards general PA.

Those participants who were members of a SVMB exercise group reported that CRT was taken into account too little (e.g. “*I quickly realised that the exercise group neglects cardiovascular training. Quite clearly. And that is almost more important than flexibility. You have to take care of your fitness yourself.” A2)*.

Furthermore, the conceptual difference between general PA and CRT seemed to be unclear to some participants. If they were asked which kind of CRT they perform, answers were very diverse, covering a range from unspecific descriptions of general, often transport-related, activities (e.g. *“I just walk a lot, I don't have a car (..) I walk relatively fast.” C2)* to detailed knowledge of CRT by ambitious athletes *(e.g. “.. if I want to improve my fitness, then I have to go beyond this limit and see what pulse rate I train at. Otherwise, I lose fitness instead of improving it.” D2*).

### Normative beliefs

For normative beliefs, no distinction between general PA and CRT was observable. Participants described several beliefs of significant others (e.g. spouses, friends) who had an influence on their motivation to comply with such beliefs. Further relevant people mentioned were other patients participating in axSpA exercise groups, the rheumatologist, and the physiotherapist. Many individuals with axSpA like to be experienced by others as being positive and sportive people (e.g. *“The doctor at the hospital said that he likes axSpA people best because they are consistently nice and positive people. They can be devastated, but as soon as they can walk again, they laugh again. People with axSpA are basically positive.” B3*). This might provoke resistance in people unable to fulfil this ideal (e.g. “*And especially when I'm in pain, I can't motivate myself to go to the gym because I'd embarrass myself. I'll just watch the others (..), in the fitness centre, everyone is watching you.” C3*). Some made a point of being independent and competent in self-management *(*e.g. *“After that, I had a physiotherapist who helped very little through physical treatment, but developed exercises with me that I can do myself at home. I don’t want her doing something with her magic hands and afterwards I feel great, I want her to show me how I can help myself. And that's the crucial thing—I don't want to spend my whole life running after a physiotherapist or a rheumatologist.” C1).* Others saw the weekly physiotherapy session as a matter of course *(*e.g. *“I see my physiotherapist once a week” E5).*

Exercising together with other people was perceived as fun and motivating. Arranging joint physical activity or exercise sessions helped to overcome the “weaker self”. The competing atmosphere of an exercise group was perceived as both motivating (e.g. “*drill and fun”,* B1; *“you can compare, you can bolster each other up”,* A4) and a deterrent (e.g. “*I like more to exercise according to my possibilities without having to justify myself”*, C5). Many participants described the axSpA exercise group as socially important (e.g. *“The social component should not be underestimated”,* C1; “*We have a good group, half of it goes for a drink after exercising”,* A5). Others preferred exercise groups not specifically designed for individuals with axSpA, because they did not need to think or talk about their condition (e.g. “*When I go to handball I'm just like any other and I don't have to deal with my back problem”, B4).*

### Control beliefs

Both, general PA and CRT, were mentioned as effective self-management strategies to control their disease *(*e.g. “*One must have axSpA under control, not the other way around*” B1). Some reported a “key experience” that helped them to realise the importance of PA (e.g. *“There is a sequence that is firmly in my memory. I could hardly move. Nevertheless, I wanted to go horse riding… when you're in so much pain, you can't move. So, I somehow got my leg up and got into the saddle. I struggled onto the horse with tears in my eyes and started riding. And the longer the horse kept moving, the better the pain got. That was a key experience for me. Although everything hurt, in the end the movement did me good.” A3*).

Regular PA and specific exercising (mostly CRT) were perceived as a matter of discipline, especially when someone did not enjoy it. Many individuals with axSpA are experts in how to integrate general PA and regular exercises into their daily routine (e.g. “*I walk the stairs, I work on the 5th floor, I walk in the morning, at noon, in the evening and in between when I have to go to the construction site.” B2)*. Almost all participants of the focus groups had examples (e.g. *“I was not born an athletic person, I had to acquire that. That I really do a lot and incorporate it into everyday life.” A1; “I have a home trainer (..) but I think riding a bike is terrible. I leave the TV on and then I know how many minutes… and then I think, that's terrible! However, I just do it then.” A3*). A positive attitude and the perceived benefits of PA and specific exercise help individuals to create time and material resources for exercising (e.g. buying a home trainer bike). Less active individuals did not describe strategies of how to integrate general PA or specific exercises into their daily routine. Some participants underlined the importance of setting an exercise goal (e.g. “*When I have a goal, I do it. Then it's fun. But I need a goal. If I don't have one, I don't do anything.” A5*). Reaching the goal reinforced their self-efficacy.

Nonetheless, the disease is associated with obstacles to exercise, since it limits the choice of sport and can also force a change in occupational career, which is sometimes hard to accept (e.g. *“I used to be an aerobics instructor (..) and had to give that up” C3*). But participants also experienced facilitators to exercise: regular participation in group exercise and the group-leading PT are often perceived as supportive (e.g. “*Physiotherapists always have a bunch of ideas [how to exercise]” B3*).

Technology-support for general PA was viewed as both motivating and demotivating. A reminder, through a tracker function that controls steps per day, was perceived as stimulating, especially for beginners or light intensity exercisers (e.g. *“I think tracking steps per day is good for a start. Everyone has a different motivation.” D2; “When I get up in the morning, I plan it (training), and when I remember it, it's in the evening. In between, the day is so packed that I forget. But when I am reminded, I incorporate it more. Because I want to do it, because I know it does me good. I do it 2 times a week … most of the time. But never without a reminder.” E2).* Others felt demotivated *(e.g. “I got a tracking watch from my wife as a birthday present. But I was so bad that I gave it back to her.” E5*).

Participants agreed that if a specific CRT goal is to be reached, technology-based pulse-control is required (e.g. “*And then there are the athletes who are training for a goal. And if you want to train properly it has to be pulse-oriented.” D3).*

## Discussion

The individuals with axSpA participating in our study showed multifaceted behavioural, normative and control beliefs concerning CRT and general PA. However, the conceptual difference between general PA and CRT, and the relevance of CRT, was unclear to some. General PA is acknowledged to have a beneficial impact on personal health and wellbeing. For most participants, general PA was part of their daily routine and was understood to be an important self-management strategy. Despite the importance of CRT in reducing the increased cardiovascular risk of people with axSpA, only a small number of participants seemed to know how to perform regular CRT and did so. The engagement in CRT requires skills and empowerment, such as detailed knowledge, social support, and a strong motivation to exercise in contempt of barriers, in particular, pain or fatigue.

Some implications can be derived from this finding for clinical practice, education, and research.

PA is behaviourally complex and increasing PA as a lifestyle change is challenging [28, 40]. Facilitating factors for individuals with axSpA include, but are not limited to, (intrinsic) motivation, good organisational conditions, improvement of disease symptoms and health [21, 41]. Barriers to an active lifestyle include, but are not limited to, low motivation, problems with timing in the daily routine, hindering disease symptoms (notably pain or fatigue) and high disease activity [21, 41, 42]. In CRT, motivation is a top facilitator and top barrier [21].

PTs, but also other health professionals, need to understand the complexity of PA behaviour and learn how to promote change using appropriate (behavioural change) techniques [43]. For instance, general practitioners and rheumatologists tend to have a long-term relationship with their patients. However, in the context of exercise prescription, general practitioners think that nurses or PTs are more skilled to promote PA [44]. Word Physiotherapy claims that physiotherapy excellence lies in the promotion, guidance, and prescription of safe PA across the life span [45]. Although this might be the case for well-informed PTs [46], there is literature describing cases of PTs with misconceptions regarding knowledge, attitudes and beliefs about PA [47], and not themselves fulfilling PA recommendations [48, 49]. Rehorn and colleagues [50] highlight that addressing individual (e.g. increasing PTs’ knowledge, skills) and organisational factors (e.g., touching upon perceived lack of time and increasing organizational support) may improve PTs’ PA promotion interventions. Verbal information on the benefits of PA and specific exercises is not usually sufficient to change behaviour. Prior to starting an exercise intervention, a PT should explore a patient’s beliefs regarding PA and exercise, detect individual barriers and define a goal, according to individual preferences and through shared decisions. Sufficient coaching, exercise guidance and correctly dosed exercise could enhance the adherence to long-term PA [51]. Our data show how diverse the understanding and personal approaches to PA and CRT can be, e.g. for some patients pain is a barrier, while for others it is the main reason for exercising. Tailored goals and patient-oriented communication strategies are especially important in handling these differences. However, as yet unpublished data on PTs working in Switzerland show that only a small number of behaviour change techniques are used, which underpins the need for the continuous training of PTs in communication and behavioural change techniques.

Patients also require further education and knowledge, since the power and effectiveness of correctly dosed exercise as medicine is still undervalued. Gossec and colleagues [52] described the belief that PA had negative effects on the disease as being associated with poor education and psychological issues, such as anxiety. Some participants in our study also mentioned their anxiety that performing PA could worsen a flare up or symptoms. However, the contrary is true. People living with axSpA benefit from both low and high intensity exercise [53–56]. PA is associated with better function and greater exercise capacity, while sedentary behaviour is associated with poor quality of life and lower exercise capacity [57].

Further research should focus on the development of strategies on how to best implement educational interventions for health professionals and people living with axSpA, and thus provide the knowledge and skills on how to promote the recommended performance of general PA and CRT [9].

The lack of distinction between the concepts of PA and exercise is common, not only in individuals with axSpA [23, 58]. If people cannot distinguish between these concepts, they see no need to engage in formal exercise and simply integrate more PA into their daily routine, increasing the duration rather than the intensity of PA. However, intensity matters in combatting the increased risk of cardiovascular disease. It is particularly important for individuals with axSpA to perform regular, moderate to vigorously-dosed CRT. Regardless of disease activity, individuals with axSpA tend to perform less outdoor activities but with higher intensity compared to healthy controls [59]. However, both aspects, outdoor activity and the intensity of PA, should be taken into account for axSpA management. Interestingly, there is some evidence to show that people are more likely to perform intense exercises outdoors, but – paradoxically—perceive it as less exhausting [60]. The natural outdoor environment generally increases physical and psychological health [61], unsurprisingly, since weather conditions are associated with outdoor PA in people with arthritis [62].

The findings of our study are in line with others [63], underpinning the conclusion that group exercise is perceived as supportive in terms of motivation (encouraging) and peer reinforcement. However, there is no evidence that group exercise is more effective than individual exercise. Group supervisors need to be aware that a “one-size fits all” approach is not useful and that the dose of exercises needs to be tailored to the fitness state of the individual [9]. Independent of setting, patients report that continual guidance and acknowledgement, and receiving advice and feedback from a supervisor is important—especially during high-intensity exercising [55]. Further research should consider how the promising area of technology-based counselling [64] could be integrated into axSpA exercise groups to promote individually-tailored CRT.

A further focus of future research could be the objective assessment of daily PA in conjunction with PA/exercise beliefs to better understand behaviour patterns. The resulting information could be used to design a composite tool to measure core attitudes and beliefs to PA/exercise in people living with axSpA. This would be useful as a standardized tool for use across studies and to develop specific interventions. Finally, it is important to investigate whether changing the attitudes and beliefs of people has a positive impact on long-term PA/exercise behaviour. In this regard, other relevant behaviour change theories [27], such as self-efficacy, should also be considered.

In terms of generalisability, the results of this study should be interpreted with caution. As commonly is the case in focus groups, participants had an interest in the topic under study [65]. Therefore, insight was gained only into the beliefs of those individuals who are interested in PA/CRT. The study results might not be representative for those individuals with no interest in PA/CRT. However, the study participants living with axSpA presented a wide range of PA behaviour, ranging from no regular PA engagement at all to exercising in a performance-oriented manner. Furthermore, the study covered the complete range of population, in terms of age, gender and membership of the SVMB exercise group, which is an important prerequisite for the generalisability of focus groups findings [66]. Additionally, since the focus groups were conducted only in the German language, generalisability is limited for those people living with axSpA in Switzerland who are not proficient in the German language.

Although the main themes of the focus groups were the barriers and facilitators to CRT and technology-based CRT, our data revealed further interesting material on the general beliefs of participants concerning CRT and PA. During analysis of results, however, it became apparent that it was unclear whether these statements referred to CRT or to general PA. In conclusion, it can be assumed that the participants’ beliefs regarding CRT and PA may differ. A detailed elaboration of these differences should be the subject of future research.

## Conclusions

People living with axSpA show multifaceted behavioural, normative and control beliefs concerning CRT and general PA that underpin the beneficial impact on personal health and wellbeing (behavioural belief). For most participants, general PA is part of their daily routine and it is understood to be an important self-management strategy (control belief). However, not all participants were aware of the importance of CRT and only a few possessed the knowledge and skills to perform CRT. PTs play a key role in PA promotion and influence normative beliefs, which could be used to a greater extent in the future to promote active lifestyle competencies in people living with axSpA. Concrete training and implementation strategies at the level of patients and PTs need to be developed in the future through further research.

## Supplementary Information

**Additional file 1.** COREQ checklist.

**Additional file 2.** Characteristics of the research team.

**Additional file 3.** Key questions of semi-structured focus group guides.

**Additional file 4: Table S3.** Results supporting citations.

## Data Availability

The datasets used and analysed during the current study are available from the corresponding author on reasonable request.

## References

[1] van der Heijde D, Ramiro S, Landewe R, Baraliakos X, Van den Bosch F, Sepriano A (2017). 2016 update of the ASAS-EULAR management recommendations for axial Spondyloarthritis. Ann Rheum Dis.

[2] Mathieu S, Gossec L, Dougados M, Soubrier M (2011). Cardiovascular profile in ankylosing spondylitis: a systematic review and meta-analysis. Arthritis Care Res (Hoboken).

[3] Nigil Haroon N, Paterson MJ, Li P, Inman RD, Haroon N (2015). SAT0272 Increased risk of vascular mortality in axial Spondyloarthritis. Ann Rheum Dis.

[4] Dagfinrud H, Kvien TK, Hagen KB (2008). Physiotherapy interventions for ankylosing spondylitis. Cochrane Database Syst Rev.

[5] Dursun N, Sarkaya S, Ozdolap S, Dursun E, Zateri C, Altan L (2015). Risk of falls in patients with ankylosing spondylitis. J Clin Rheumatol.

[6] Sahin N, Ozcan E, Baskent A, Karan A, Kasikcioglu E (2011). Muscular kinetics and fatigue evaluation of knee using by isokinetic dynamometer in patients with ankylosing spondylitis. Acta Reumatol Port.

[7] Peters MJ, Visman I, Nielen MM, Van Dillen N, Verheij RA, van der Horst-Bruinsma IE (2010). Ankylosing spondylitis: a risk factor for myocardial infarction?. Ann Rheum Dis.

[8] Noureldin B, Barkham N (2018). The current standard of care and the unmet needs for axial Spondyloarthritis. Rheumatology (Oxford).

[9] Rausch Osthoff AK, Niedermann K, Braun J, Adams J, Brodin N, Dagfinrud H (2018). 2018 EULAR recommendations for physical activity in people with inflammatory arthritis and osteoarthritis. Ann Rheum Dis.

[10] Rausch Osthoff AK, Juhl CB, Knittle K, Dagfinrud H, Hurkmans E, Braun J (2018). Effects of exercise and physical activity promotion: meta-analysis informing the 2018 EULAR recommendations for physical activity in people with rheumatoid arthritis, spondyloarthritis and hip/knee osteoarthritis. RMD Open.

[11] Services USDoHaH (2020). Physical activity guidelines for Americans.

[12] Nystoriak MA, Bhatnagar A (2018). Cardiovascular effects and benefits of exercise. Front Cardiovasc Med.

[13] Sveaas SH, Smedslund G, Hagen KB, Dagfinrud H (2017). Effect of cardiorespiratory and strength exercises on disease activity in patients with inflammatory rheumatic diseases: a systematic review and meta-analysis. Br J Sports Med.

[14] Sveaas SH, Berg IJ, Provan SA, Semb AG, Hagen KB, Vollestad N, Fongen C, Olsen IC, Michelsen A, Ueland T, Aukrust P, Kvien TK, Dagfinrud H (2014). Efficacy of high intensity exercise on disease activity and cardiovascular risk in active axial Spondyloarthritis: a randomized controlled pilot study. PLoS ONE.

[15] Ward MM, Deodhar A, Gensler LS, Dubreuil M, Yu D, Khan MA (2019). 2019 Update of the American College of Rheumatology/Spondylitis Association of America/Spondyloarthritis research and treatment network recommendations for the treatment of ankylosing spondylitis and nonradiographic axial Spondyloarthritis. Arthritis Rheumatol (Hoboken, NJ).

[16] O'Dwyer T, O'Shea F, Wilson F (2015). Decreased physical activity and cardiorespiratory fitness in adults with ankylosing spondylitis: a cross-sectional controlled study. Rheumatol Int.

[17] van Genderen S, Boonen A, van der Heijde D, Heuft L, Luime J, Spoorenberg A (2015). Accelerometer quantification of physical activity and activity patterns in patients with ankylosing spondylitis and population controls. J Rheumatol.

[18] Rausch Osthoff AK, van der Giesen F, Meichtry A, Walker B, Van Gaalen F, Goekoop-Ruitermans YPM (2019). The perspective of people with axial Spondyloarthritis regarding physiotherapy: room for the implementation of a more active approach. Rheumatol Adv Pract.

[19] Brophy S, Cooksey R, Davies H, Dennis MS, Zhou SM, Siebert S (2013). The effect of physical activity and motivation on function in ankylosing spondylitis: a cohort study. Semin Arthritis Rheum.

[20] Rasmussen JO, Primdahl J, Fick W, Bremander A (2020). Physical activity in people with axial Spondyloarthritis and the impact of overall attitudes, barriers, and facilitators: a cross-sectional study. Musculoskelet Care.

[21] Niedermann K, Nast I, Ciurea A, Vliet Vlieland T, van Bodegom-Vos L (2019). Barriers and facilitators of vigorous cardiorespiratory training in axial Spondyloarthritis: surveys among patients, physiotherapists, and rheumatologists. Arthritis Care Res.

[22] Verhagen E, Engbers L (2009). The physical therapist's role in physical activity promotion. Br J Sports Med.

[23] Dasso NA (2019). How is exercise different from physical activity? A concept analysis. Nurs Forum.

[24] Garber CE, Blissmer B, Deschenes MR, Franklin BA, Lamonte MJ, Lee IM (2011). American College of Sports Medicine position stand. Quantity and quality of exercise for developing and maintaining cardiorespiratory, musculoskeletal, and neuromotor fitness in apparently healthy adults: guidance for prescribing exercise. Med Sci Sports Exerc.

[25] Caspersen CJ, Powell KE, Christenson GM (1985). Physical activity, exercise, and physical fitness: definitions and distinctions for health-related research. Public Health Rep.

[26] Knittle K, De Gucht V, Maes S (2012). Lifestyle- and behaviour-change interventions in musculoskeletal conditions. Best Pract Res Clin Rheumatol.

[27] Larkin L, Kennedy N, Gallagher S (2015). Promoting physical activity in rheumatoid arthritis: a narrative review of behaviour change theories. Disabil Rehabil.

[28] Biddle SJH, Mutrie N (2001). Psychology of physical activity.

[29] Kan MPH, Fabrigar LR, Zeigler-Hill V, Shackelford TK (2017). Theory of planned behavior. Encyclopedia of personality and individual differences.

[30] Sandelowski M (2000). Focus on research methods: whatever happened to qualitative description?. Res Nurs Health.

[31] Sandelowski M (2010). What’s in a name? Qualitative description revisited. Res Nurs Health.

[32] Kitzinger J, Holloway I (2005). Focus group research: using group dynamics to explore perceptions, experiences and understandings. Qualitative research in health care.

[33] Kitzinger J (1995). Qualitative research: introducing focus group. Br Med J.

[34] Rausch Osthoff AK, Vliet Vlieland TPM, Meichtry A, van Bodegom-Vos L, Topalidis B, Buchi S (2022). Lessons learned from a pilot implementation of physical activity recommendations in axial Spondyloarthritis exercise group therapy. BMC Rheumatol.

[35] General Assembly of the World Medical A (2014). World Medical Association Declaration of Helsinki: ethical principles for medical research involving human subjects. J Am Coll Dent.

[36] Tong A, Sainsbury P, Craig J (2007). Consolidated criteria for reporting qualitative research (COREQ): a 32-item checklist for interviews and focus groups. Int J Qual Health Care.

[37] Craig CL, Marshall AL, Sjostrom M, Bauman AE, Booth ML, Ainsworth BE (2003). International physical activity questionnaire: 12-country reliability and validity. Med Sci Sports Exerc.

[38] Steinke I, Flick U, von Kardoff E, Steinke I (2009). Gütekritierien qualitativer Forschung. Qualitative Forschung Ein Handbuch.

[39] GmbH SSD. Atlas.ti qualitative data analysis, 7–9th edn. 2021. https://atlasti.com/.

[40] Gabriel KKP, Morrow JR, Woolsey ALT (2012). Framework for physical activity as a complex and multidimensional behavior. J Phys Act Health.

[41] Liu SH, Morais SA, Lapane KL, Kay J (2020). Physical activity and attitudes and perceptions towards physical activity in patients with spondyloarthritis: a systematic review. Semin Arthritis Rheum.

[42] Fongen C, Sveaas SH, Dagfinrud H (2015). Barriers and facilitators for being physically active in patients with ankylosing spondylitis: a cross-sectional comparative study. Musculoskelet Care.

[43] Kunstler BE, Cook JL, Freene N, Finch CF, Kemp JL, O'Halloran PD (2018). Physiotherapists use a small number of behaviour change techniques when promoting physical activity: a systematic review comparing experimental and observational studies. J Sci Med Sport.

[44] Persson G, Brorsson A, Ekvall Hansson E, Troein M, Strandberg EL (2013). Physical activity on prescription (PAP) from the general practitioner's perspective—a qualitative study. BMC Fam Pract.

[45] World_Physiotherapy. Physical therapists as exercise and physical activity experts across the life span. Policy statement. World Confederation for Physical Therapy; 2019. https://world.physio/policy/ps-exercise-experts.

[46] Shirley D, van der Ploeg HP, Bauman AE (2010). Physical activity promotion in the physical therapy setting: perspectives from practitioners and students. Phys Ther.

[47] Mounton A, Mugnier B, Demoulin C, Cloues M (2014). Physical therapists’ knowledge, attitudes, and beliefs about physical activity: a prerequisite to their role in physical activity promotion?. J Phys Ther Educ.

[48] Neil-Sztramko SE, Ghayyur A, Edwards J, Campbell KL (2017). Physical activity levels of physiotherapists across practice settings: a cross-sectional comparison using self-report questionnaire and accelerometer measures. Physiother Can.

[49] Lowe A, Littlewood C, McLean S, Kilner K (2017). Physiotherapy and physical activity: a cross-sectional survey exploring physical activity promotion, knowledge of physical activity guidelines and the physical activity habits of UK physiotherapists. BMJ Open Sport Exerc Med.

[50] Rethorn ZD, Covington JK, Cook CE, Bezner JR (2022). Physical therapists' knowledge, skills, beliefs, and organizations impact physical activity promotion: a systematic review and meta-analysis. Phys Ther.

[51] Bilberg A, Dagfinrud H, Sveaas SH (2022). Supervised intensive exercise strengthen exercise health beliefs in patients with axial Spondyloarthritis: a multicentre randomized controlled trial. Arthritis Care Res..

[52] Gossec L, Berenbaum F, Chauvin P, Hudry C, Cukierman G, de Chalus T (2018). Development and application of a questionnaire to assess patient beliefs in rheumatoid arthritis and axial Spondyloarthritis (vol 37, pg 2649, 2018). Clin Rheumatol.

[53] O'Dwyer T, McGowan E, O'Shea F, Wilson F (2016). Physical activity and exercise: perspectives of adults with ankylosing spondylitis. J Phys Act Health.

[54] Demmelmaier I, Lindkvist A, Nordgren B, Opava CH (2015). "A gift from heaven" or "This was not for me". A mixed methods approach to describe experiences of participation in an outsourced physical activity program for persons with rheumatoid arthritis. Clin Rheumatol.

[55] Bilberg A, Sveaas SH, Dagfinrud H, Mannerkorpi K (2020). How do patients with axial Spondyloarthritis experience high-intensity exercise?. Acr Open Rheumatol.

[56] Sveaas SH, Bilberg A, Berg IJ, Provan SA, Rollefstad S, Semb AG (2020). High intensity exercise for 3 months reduces disease activity in axial Spondyloarthritis (axSpA): a multicentre randomised trial of 100 patients. Br J Sports Med.

[57] Coulter EH, McDonald MT, Cameron S, Siebert S, Paul L (2020). Physical activity and sedentary behaviour and their associations with clinical measures in axial Spondyloarthritis. Rheumatol Int.

[58] Hurley M, Dickson K, Hallett R, Grant R, Hauari H, Walsh N (2018). Exercise interventions and patient beliefs for people with hip, knee or hip and knee osteoarthritis: amixedmethods review. Cochrane Database Syst Rev.

[59] Fongen C, Halvorsen S, Dagfinrud H (2013). High disease activity is related to low levels of physical activity in patients with ankylosing spondylitis. Clin Rheumatol.

[60] Focht BC (2009). Brief walks in outdoor and laboratory environments: effects on affective responses, enjoyment, and intentions to walk for exercise. Res Q Exerc Sport.

[61] Aliyas Z (2021). Physical, mental, and physiological health benefits of green and blue outdoor spaces among elderly people. Int J Environ Health Res.

[62] Timmermans EJ, van der Pas S, Dennison EM, Maggi S, Peter R, Castell MV (2016). The influence of weather conditions on outdoor physical activity among older people with and without osteoarthritis in 6 European countries. J Phys Act Health.

[63] Hilberdink B, van der Giesen F, Vliet Vlieland T, Nijkamp M, van Weely S (2020). How to optimize exercise behavior in axial Spondyloarthritis? Results of an intervention mapping study. Patient Educ Couns.

[64] Li LDC, Feehan LM, Xie H, Lu N, Shaw CD, Gromala D (2020). Efficacy of a physical activity counseling program with use of a wearable tracker in people with inflammatory arthritis: a randomized controlled trial. Arthritis Care Res.

[65] Acocella I (2012). The focus groups in social research: advantages and disadvantages. Qual Quant.

[66] Bloor M, Frankland J, Thomas M, Robson K (2001). Conclusions. Focus groups in social research. Introducing qualitative methods.

