# Supplementary material for: Understanding beliefs related to physical activity in people living with axial Spondyloarthritis: a theory-informed qualitative study
Source: BMC Rheumatol. 2022 Jul 25;6:40. doi: 10.1186/s41927-022-00270-2 (PMC9310396; doi:10.1186/s41927-022-00270-2)
Supplement: Supplementary file 1 — Additional file 1. COREQ checklist. [file 41927_2022_270_MOESM1_ESM.docx]

**Supplement A1.** Consolidated criteria for reporting qualitative studies (COREQ^1^): 32- intem checklist

| **No.** | **Item** | **Section** | **Page** |
| --- | --- | --- | --- |
| 1 | Interviewer | Supplement A.2 | 1 |
| 2 | Credentials | Supplement A.2 | 1 |
| 3 | Occupation | Supplement A.2,  Methods, data analysis | 1  8 |
| 4 | Gender | Supplement A.2 | 1 |
| 5 | Experience and training | Supplement A.2 | 1 |
| 6 | Relationship established | Methods, procedures | 8 |
| 7 | Participant knowledge of the interviewer | Methods, procedures | 8 |
| 8 | Interviewer characteristics | Supplement A.2 | 1 |
| 9 | Methodological orientation and Theory | Methods, design | 6 |
| 10 | Sampling | Methods, procedures | 7 |
| 11 | Method of approach | Methods, procedures | 8 |
| 12 | Sample size | Table 1 | 7 |
| 13 | Non-participation | Methods, procedures | 7 |
| 14 | Setting of data collection | Methods, procedures | 8 |
| 15 | Presence of non-participants | Methods, procedures | 8 |
| 16 | Description of sample | Table 1 | 7 |
| 17 | Interview guide | Supplement B | 1-2 |
| 18 | Repeat interviews | N/A |  |
| 19 | Audio/visual recording | Methods, Data analysis | 8 |
| 20 | Field notes | Methods, Data analysis | 8 |
| 21 | Duration | Methods, procedures | 8 |
| 22 | Data saturation | Discussion | 17 |
| 23 | Transcripts returned | N/A |  |
| 24 | Number of data coders | Methods, Data analysis | 8 |
| 25 | Description of the coding tree | Methods, Table 2 | 9 |
| 26 | Derivation of the themes | Methods, Data analysis | 8 |
| 27 | Software | Methods, Data analysis | 8 |
| 28 | Participant checking | Methods, Measures of trustworthiness | 9 |
| 29 | Quotations presented | Supplement C and  Results | 10-14 |
| 30 | Data and findings consistent | Results | 10-14 |
| 31 | Clarity of major themes | Results | 10-14 |
| 32 | Clarity of minor themes | Results | 10-14 |

^1^ Tong A, Sainsbury P, Craig J. Consolidated criteria for reporting qualitative research (COREQ): 28 a 32-item checklist for interviews and focus groups. Int J Qual Health Care. 2007;19(6):349- 29 57.doi:10.1093/intqhc/mzm042
