# Supplementary material for: Understanding beliefs related to physical activity in people living with axial Spondyloarthritis: a theory-informed qualitative study
Source: BMC Rheumatol. 2022 Jul 25;6:40. doi: 10.1186/s41927-022-00270-2 (PMC9310396; doi:10.1186/s41927-022-00270-2)
Supplement: Supplementary file 2 — Additional file 2. Characteristics of the research team. [file 41927_2022_270_MOESM2_ESM.docx]

**Supplement A2: characteristics of the research team**

| **Characteristic** | **Description** |
| --- | --- |
| Credentials | IN and KN hold a doctoral degree and a professorship. AR has a Master of Science degree and is a doctoral candidate. |
| Occupation | IN is psychologist, KN and AR are physiotherapists. All three work as researchers at Zurich University of Applied Sciences, Institute for Physiotherapy. |
| Gender | IN, KN, AR are female. |
| Experience and training | IN, KN, AR are authors of multiple quantitative and qualitative publications. IN is expert in qualitative research methods with many years of experience in conducting and analysing focus groups. |
| Interviewer characteristics | IN, KN and AR are interested in the promotion of physical activity in people with chronic, rheumatic conditions. A premise for the promotion of physical activity is understanding the target group. |
