# Supplementary material for: Understanding beliefs related to physical activity in people living with axial Spondyloarthritis: a theory-informed qualitative study
Source: BMC Rheumatol. 2022 Jul 25;6:40. doi: 10.1186/s41927-022-00270-2 (PMC9310396; doi:10.1186/s41927-022-00270-2)
Supplement: Supplementary file 3 — Additional file 3. Key questions of semi-structured focus group guides. [file 41927_2022_270_MOESM3_ESM.docx]

**Understanding beliefs related to physical activity in people living with axial Spondyloarthritis - a theory-informed qualitative study**

**Supplement B: Key questions of semi-structured focus group guides**

| Focus group (FG) phase | FGs on importance of physical activity (PA) and barriers and facilitators to cardiorespiratory training (CRT) | FGs on physical activity behaviour and technology-based cardiorespiratory training (CRT) |  |
| --- | --- | --- | --- |
| Introduction | Please introduce yourself with your name, age and briefly tell us something about yourself (e.g. your (former) professional activity, favourite leisure activities). | Please introduce yourself with your name, age and briefly tell us something about yourself (e.g. your (former) professional activity, favourite leisure activities). |  |
| Main phase:  part 1 | How would you currently describe your state of health?  Are you actively doing something for your health/ well-being? If yes, please describe your activities and how they affect your health/ well-being.  What helps you most in connection with your illness? | How would you currently describe your state of health?  Are you actively doing something for your health/ well-being? If yes, please describe your activities and how they affect your health/ well-being.  What helps you most in connection with your illness? |  |
| Main phase:  part 2 | How important is physical activity to you and why?  In the past and currently?  How would you describe your physical activity behaviour?  What do you think about sport or training, what do the terms mean to you? | How important is CRT to you? Do you regularly perform CRT? What – how often – how long – when; if not, why?  Do you know exercise principles? What impact has CRT made on your disease activity? |  |
| Main phase:  part 3 | What do you think about cardiovascular training?  Do you do any form of CRT yourself? What, how often and why (or why not)?  What influence could CRT have on the health of people living with Axial Spondyloarthritis? | Do you perform technology-based CRT? What are your experiences? What devices and functions do you use? What are facilitators? What hampers the use of technology? |  |
| Main phase:  part 4 | If member of the SVMB Exercise Groups:  What is your experience with the SVMB exercise group? | Individual support: Do you need counselling to start/optimize your CRT? Why yes/not? What setting (e.g. frequency, web-based) would you prefer?  Would you like to receive CRT reminders (e.g. sms/e-mail)? Why yes/not? Frequency?  How much money would you want to spend per month/year for such a service (e.g. technology-based exercising, coaching)? | |
| Main phase:  part 5 | To maintain health, it is recommended to exercise at least 150 minutes/week.  If you achieve this level: what helps you to achieve or maintain this level?  If you exercise less or not at all: what is the reason? What could support you to exercise more often? | How can the SVMB support you in CRT? do you have expectations? Individual level and group exercise level | |
| Conclusion | If you look back at the discussion...  What surprised/astonished you?  What do you think were the important elements of today's discussion?  What other topics should be considered? | If you look back at the discussion...  What surprised/astonished you?  What do you think were the important elements of today's discussion?  What other topics should be considered? | |
