# Supplementary material for: Understanding beliefs related to physical activity in people living with axial Spondyloarthritis: a theory-informed qualitative study
Source: BMC Rheumatol. 2022 Jul 25;6:40. doi: 10.1186/s41927-022-00270-2 (PMC9310396; doi:10.1186/s41927-022-00270-2)
Supplement: Supplementary file 4 — Additional file 4: Table S3. Results supporting citations. [file 41927_2022_270_MOESM4_ESM.docx]

**Supplement B**

Table 3: Results supporting citations

| **Category** | **Theme** | | | **Quotation ID** | **Quotation** |
| --- | --- | --- | --- | --- | --- |
| **Attitudes towards behaviour** | | | | | |
| Conceptualization | PA and CRT | | | Qa_Concept_1  Qa_concept_2  Qa_concept_3  Qa_concept_4  Qa_concept_5 | *“I just walk a lot, I don't have a car (..) I walk relatively fast.” C2*  *“We always go for a walk on the (xy) mountain. Well, from time to time we walk a little faster. But not jogging.” A4*  *“..and then I get into such a [heart frequency] range that I notice ..I have a high pulse (..) and I am sweating profusely.” A3*  *“.. if I want to improve my fitness, then I have to go beyond this limit and see what pulse rate I train at. Otherwise, I lose fitness instead of improving it.” D2*  *“If you want to train correctly, then it simply has to be pulse-oriented..(..) you get much more from your training” D3* |
|  | Perceived relevance | | | Qa_SC_1  Qa_SC_2  Qa_SC_3 | *“I miss something if I cannot exercise”, C5*  *“I am very sporty” C6*  *“When I haven't done the exercises, I feel it. Then I do it by necessity.” C2* |
|  | Lack of knowledge | | | Qa_CRT_1  Qa_CRT_2 | *“Why are people so into cardiorespiratory training recently, it always comes up..?” B3*  *“Does that [CRT] also have a positive effect on a flare up, on the inflammation, .. ?” B1* |
|  | CRT in exercise group | | | Qa_CRT_group_1 | *“I quickly realised that the exercise group neglects cardiovascular training. Quite clearly. And that is almost more important than flexibility. You have to take care of your fitness yourself”.A2* |
| Exercise benefits | Physical | | | Qa_ben_phys_1  Qa_ben_phys_2 | *“I think axSpA also has advantages. You stay active” C6*  *“I'm tired, anyway, but better tired. That's for sure.” D4* |
|  | Psychological | | | Qa_ben_psy_1  Qa_ben_psy_2  Qa_ben_psy_3 | *“Exercising is fun, I love biking” A5*  *“I feel better, I feel more comfortable” C2*  *“I also feel better when I move. You have the same pain, but then you know it's just a good feeling when you don't do anything and you have pain, then it's more of a negative feeling. (..) I agree, physical activity is simply most important” D3* |
|  | Social | | | Qa_ben_soc_1 | *“For us, group therapy is first and foremost a social gathering. More than anything else. That's the beauty of/good thing about group therapy”. E6* |
| Exercise risks | Negative associations | | | Qa_risk_1  Qa_risk_2 | *“It takes a lot of effort for me to learn how to ski, I am very afraid I might fall.”C1*  *“When I listen to my pain, I don't do sport anymore.” B4* |
| **Normative beliefs** | | | | | |
| Significant others | axSpA Peers | Mutual commitment | | Qn_peers_1  Qn_peers_2 | *“I think the urge to exercise is typical for people with axSpA” B3*  *“Exercise is good, we can almost not do too much” B2* |
|  |  | Performance pressure | | Qn_peers_3  Qn_peers_4 | *“I have a guilty conscience. When I hear this... I think I'll pack my things and leave. I'm far away from the others. (..). I don't have the time. I work an average of 12 hours a day, have 3 children at home. When I get home, I can't go for a quick bike ride. That's how it is.” TN5*  *“And especially when I'm in pain, I can't motivate myself to go to the gym because I'd embarrass myself. I'll just watch the others and do it like that. Or, in the fitness centre, everyone is watching you.”C3* |
|  | Rheumatologist | Image of people with axSpA | | Qn_MD_1 | *“The doctor at the hospital said that he likes axSpA people best because they are consistently nice and positive people. They can be devastated, but as soon as they can walk again, they laugh again. People with axSpA are basically positive.” B3* |
|  | Physiotherapist | | | Qn_PT_1  Qn_PT_2  Qn_PT_3 | *“After that, I had a physiotherapist who helped very little through physical treatment but developed exercises with me that I can do myself at home. I don’t what her doing something with her magic hands and afterwards I feel great, but I want her to show me how I can help myself. And that's the crucial thing - I don't want to spend my whole life running after a physiotherapist or a rheumatologist.” C1*  *“I see my physical therapist once a week” E5*  *“I hear from other patients that they mainly go to the massage, but that is of course no use. It is, of course, pleasant.” E6* |
| **Control beliefs** | | | | | |
| Daily routine | Discipline, tricks | | | Qc_daily_1  Qc_daily_2  Qc_daily_3  Qc_daily_4  Qc_daily_5 | *“So, what I try to incorporate in everyday life is, uh, pinch buttocks (..), when you're standing at the cash register somewhere and waiting. That is simply an exercise that really strengthens the buttock muscles. So that's what I'm trying to do all the time (..) I learned that in gymnastics. Somehow it is very important for the body tension. So that's something I do regularly.” B1*  *“When I got the red calendar, the gymnastics calendar at the patient training back then, that's my alpha and omega... that's the 10 to 10 training...(..) when 10 to 10 (news on television) comes, three times a week, that's my gymnastics calendar.” D1*  *“When I'm on the cross trainer I have to force myself to watch something on TV to do it at all” A5*  *“I was not born an athletic person, I had to acquire that. That I really do a lot and incorporate it into everyday life.” A1*  *“I have a home trainer (..) but I think riding a bike is terrible. I leave the TV on and then I know how many minutes... and then I think, that's terrible! However, I just do it then.” A3* |
|  | Goal setting | | | Qc_goal_1  Qc_goal_2  Qc_goal_3 | *“The small triathlon is the goal I want to do, not that I have done it before, but just to overcome the physical barriers. You can tell I'm not a competitive athlete, but I can do it.” C1*  *“When I have a goal, I do it. Then it's fun. But I need a goal. If I don't have one, I don't do anything.” A5*  *“Jogging didn't do me so well, always tension in my neck. Still, it's just fun to know that I did the [5km run]. I really ran”. A3* |
| Key experience | Key experience | | | Qc_key_1 | *“There is a sequence that is firmly in my memory. I could hardly move. Nevertheless, I wanted to go riding... when you're in so much pain, you can't move. So, I somehow got my leg up and got into the saddle. I struggled onto the horse with tears in my eyes and started riding. And the longer the horse kept moving, the better the pain got. That was a key experience for me. Although everything hurt, in the end the movement did me good.” A3* |
| Limitation due to disease | Limitations | | | Qc_limit_1  Qc_limit_2  Qc_limit_3  Qc_limit_4  Qc_limit 5 | *“I used to be an aerobics instructor (..) and had to give that up” C3*  *“AxSpA group exercising is too hard for me. (..) I can't do the exercises. I can't stretch my leg to the ceiling and guide my arm. I have no strength.” B3*  *“That's quite a challenge to overcome because exercise is good for you, and the body is like, no, I have pain, I don't like it, and that's what I find so difficult (..) you have to find a way, what is better - just stay at home and having pain, because the other thing is that when I start sport and I'm fully into it, because with all the adrenaline and so on I don't have pain (..) , but it just comes back again. And that's really difficult for me, to decide when listening to the pain and when I have to do the same sports. But if I'm always in pain, then I don't do any sport.” B4*  *So sometimes it's just the absolute tiredness that overcomes you. So, when there's an inflammation, it's just that I... how should I put it... I don't have any energy. It's like the battery is completely empty. And I can always feel that with me when there's another inflammation. And then I have to overcome it, to pull myself together. Just the effort.” D4*  *“But basically, I would like to exercise like I used to, but that's not possible. I'm not allowed to do contact sports anymore.” B1* |
| Pain control |  | | | Qc_pain_1  Qc_pain_2  Qc_pain :3  Qc_pain_4 | *“If I (...) something gets stuck in the rib joint or something is blocked, then I put my mat on the floor and start doing exercises (...). As a person with axSpA, you know over the years what you have to do to help yourself (...). You don't always have a therapist at your disposal, right..?” C6*  *“In the evening (after work) I go for a walk and then I feel better right away.” C2*  *“Sometimes you had pain (during training), but then you just ignored it. You have pain anyway. But I think it's something really important. (...) those who are always moving, running or doing something, their fitness condition is completely different than when they do nothing.” A2*  *“You have to tell people, if you don’t exercise, pain won’t stop” D2* |
| Daily living | Exercise requirements | | | Qc_DL_1  Qc_DL_2 | *“I used to be an aerobics instructor (..) and had to give that up” C3*  *“..that's why I give (sport) courses. I need pressure. I have to have a certain basic fitness. Otherwise, I'm no longer credible. And then I have to. But if I didn't have that, I'd probably be lying around twice a week. I'm also always tired. I find the tiredness the worst thing of all about the illness.” D3* |
|  | Exercise opportunities | | | Qc_DL_3 | *“I walk the stairs, I work on the 5th floor, I walk in the morning, at noon, in the evening and in between when I have to go to the construction site.” B2* |
|  | SVMB supported | | | Qc_DL_4 | *“That's why I think it's a nice offer from the SVMB that I can just go and try group exercising, and that I'm among my peers, and I think that's the only way to motivate people. However, you cannot talk about sports, because they have a completely different agenda to the one I have always had.” C5*  *“I don't think that you go to the axSpA group to do competitive sports.”C1* |
|  | Expectations of course instructors | | | Qn_PT_4 | *“Physical therapists always have a bunch of ideas [how to exercise]” B3* |
| Technology- based CRT | Impact on motivation | | negative | Qc_tech_1  Qc_tech_2 | *“I got a tracking watch from my wife as a birthday present. But I was so bad that I gave it back to her.” E5*  *“Now that I'm a bit depressed and lacking in drive, if I had to see in figures how unfit I am, that would demotivate me. Then, I wouldn't do anything at all. (...) That's why, when I exercise, I don't want to see stuff like heart rate. It would make me nervous and demotivate me.” D4* |
|  |  |  | positive | Qc_tech_3  Qc_tech_4 | *“I think, tracking steps per day is good for a start. Everyone has a different motivation.” D2*  *“When I get up in the morning, I plan it (training), and when I remember it, it's in the evening. In between, the day is so full that I forget. But when I am reminded, I incorporate it more. Because I want to do it, because I know it does me good. I do it 2 times a week ... most of the time. But not at all without a reminder.” E2* |
|  | Pulse-controlled CRT | | | Qc_tech_5  Qc_tech_6 | *“For me, the heart rate monitor is a control instrument, I feel myself but if I'm not sure, I check my watch (...)” D1*  *“People who want to exercise but don't want to train towards a goal, they don't necessarily need pulse-oriented training. In my eyes, a fitness tracker is much better where you can see... there's a bar, when the bar is full, then I know I've taken my 10,000 steps. And that's enough for most people, isn't it? And then there are the athletes who are training for a goal. And if you want to train properly it has to be pulse-oriented.” D3* |

Note: Quotations might be allocated to several themes. However, each has been depicted to the primary theme.

Abbreviations: CRT = cardiorespiratory training

Explanation of IDs: A= active, but not in SVMB exercise group; B = less active individuals and not in SVMB exercise group; C = active and in SVMB exercise group; A-C participated in focus groups on barriers and facilitators to CRT. D and E participated in focus groups on technology-based CRT.
